# Exploring the Mechanism of Postpartum Cognitive Dysfunction in a Preeclampsia Rat Model via Multimodal Imaging

**DOI:** 10.1096/fj.202601527R

**Published:** 2026-09-28

**Authors:** Yong Xia, Junguo Zhao, Bo Gao

**Affiliations:** ^1^ Department of Radiology The Affiliated Hospital of Guizhou Medical University Guiyang Guizhou China; ^2^ Department of Radiology Guizhou Hospital of The First Affiliated Hospital of Sun Yat‐sen University (FAH‐SYSU) Gui'an New Area Guizhou China; ^3^ Key Laboratory of Brain Imaging Guizhou Medical University Guiyang Guizhou China

**Keywords:** cognitive function, magnetic resonance imaging, microvascular perfusion, pravastatin, preeclampsia

## Abstract

Preeclampsia (PE) affects 3%–5% of pregnancies worldwide and is a leading cause of maternal/perinatal mortality, yet its etiology remains unknown. We established a PE rat model using L‐NAME to investigate postpartum cognitive dysfunction and its mechanisms, evaluated brain microstructural changes with MRI, explored correlations between MRI parameters and cognitive impairment, and assessed pravastatin's therapeutic effects. Sixty‐three pregnant rats were divided into normal pregnant (NP), PE, and pravastatin‐treated (PRA) groups, with three postpartum subgroups (10 days, 1 month, 3 months; *n* = 7 each). Morris water maze, HE/Nissl staining, transmission electron microscopy, and multimodal MRI (DTI, IVIM, DCE‐MRI) were performed. ROIs included anterior/posterior cortex and hippocampus. Pearson correlation was used to relate MRI parameters to escape latency. (1) L‐NAME induced a PE‐like phenotype, alleviated by pravastatin. (2) Cognitive impairment appeared at 10 days postpartum and worsened over time. (3) FA, D, and f values decreased while Ktrans increased in the PE group (*p* < 0.05), changes that progressed over time and were abrogated by pravastatin. (4) FA and D were negatively correlated with escape latency, whereas Ktrans showed a positive correlation. (5) Neuronal degeneration, astrocyte activation, and BBB disruption in the PE group were ameliorated by pravastatin. PE model rats developed progressive postpartum cognitive dysfunction, likely due to neuronal degeneration, glial activation, and increased BBB permeability. Pravastatin partially alleviated these pathological and cognitive changes. Multimodal MRI effectively detected alterations in brain microstructure, microvascular perfusion, and BBB, consistent with histopathological findings.

## Graphical abstract

This schematic illustrates an L‐NAME‐induced preeclampsia (PE) rat model generated via L‐NAME administration to pregnant dams, wherein Morris water maze assessments confirmed progressive postpartum cognitive dysfunction that emerged at 10 days post‐partum and deteriorated further at 1 and 3 months post‐partum. Multimodal magnetic resonance imaging (MRI), including diffusion tensor imaging (DTI), intravoxel incoherent motion (IVIM), and dynamic contrast‐enhanced (DCE) MRI, was applied to systematically characterize PE‐related cerebral pathological alterations: DTI uncovered neuronal degeneration, IVIM detected diminished microvascular density (MVD), and DCE‐MRI demonstrated blood–brain barrier (BBB) breakdown. Collectively, this work validates multimodal MRI as a robust imaging platform to evaluate cerebral microstructural remodeling, microvascular perfusion deficits, and BBB injury in PE rats, and further highlights that pravastatin intervention effectively rescues PE‐related pathological phenotypes and cognitive impairments.

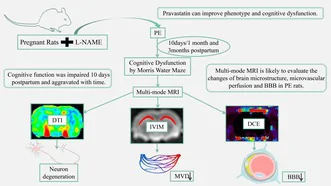

## 1 Introduction

Preeclampsia (PE) is a serious complication of pregnancy involving multiple organs and systems that occurs in 3%–5% of total pregnancies worldwide [1]. It is an important cause of maternal perinatal mortality and is characterized by the occurrence of hypertension and proteinuria after 20 weeks of gestation; moreover, it may be associated with dysfunction of other organs and disturbances in placental development [2]. The etiology and pathogenesis of PE are unknown, with the most generally accepted theory being endothelial dysfunction.

Nitro‐L‐arginine methyl ester (L‐NAME) is widely used to establish PE models [3], characterized by spiral artery remodeling abnormalities, angiogenesis disorders, and inflammation [4, 5]. L‐NAME induces dose‐dependent hypertension and proteinuria in pregnant rats [6] and increases maternal and perinatal mortality [7]. PE causes lasting brain pathological changes, serving as a risk marker for early cerebrovascular damage [8]. After childbirth, PE patients may develop intracerebral lesions and cognitive dysfunction [9], potentially due to blood–brain barrier disruption by placental antiangiogenic factors and proinflammatory cytokines, leading to long‐term brain structural and functional changes. Women with a history of PE have a > 3‐fold higher risk of vascular dementia later in life [10]. A meta‐analysis revealed a significant link between PE and subjective cognitive impairment [11].

Magnetic resonance imaging (MRI) is widely used to study brain structure and function [12]; however, few researchers have applied it to evaluate PE‐related cognitive dysfunction in PE models [13, 14]. We hypothesized that PE‐induced postpartum cognitive dysfunction is associated with region‐specific alterations in brain microstructure (assessed by FA), microvascular perfusion (assessed by D*), and BBB integrity (assessed by Ktrans). The anterior cortex, posterior cortex, and hippocampus were selected as ROIs since the prefrontal cortex plays a critical role in spatial working memory and executive function, the posterior cortex integrates visuospatial information, and the hippocampus is essential for learning and memory consolidation. These regions have been shown to be vulnerable to hypertensive and inflammatory injury in PE models. Therefore, we used multimodal MRI to assess brain microstructural changes in PE model rats cross‐sectionally and longitudinally, comparing the results with cognitive and pathological examinations to explore the mechanisms of PE‐induced cognitive dysfunction.

PE is treated primarily symptomatically (e.g., antihypertensives, magnesium sulfate), but the most effective intervention is fetal delivery. Low‐dose aspirin is recommended for prophylaxis in high‐risk women [15], yet no drug effectively treats PE or improves its prognosis.

Statins improve endothelial function and reduce inflammation [16] by increasing nitric oxide bioavailability, promoting reendothelialization, reducing oxidative stress, and suppressing inflammatory responses [17]. Although pravastatin is no longer absolutely contraindicated in pregnancy, it is not recommended for routine clinical use due to limited safety data and lack of approval for PE treatment. Preclinical and clinical studies have reported that pravastatin reverses pregnancy‐specific angiogenic disorders, restores endothelial function, and prevents oxidative/inflammatory damage in PE [18]; however, its efficacy in human trials remains unverified.

In this study, a PE rat model was established with L‐NAME. At 10 days, 1 month, and 3 months postpartum, we performed the Morris water maze test, followed by multimodal MRI. Regions of interest in the anterior cortex, posterior cortex, and hippocampus were analyzed for correlations between MRI parameters and cognitive dysfunction indicators. We also discuss the mechanisms of postpartum cognitive dysfunction in PE and the value of MRI for cognitive evaluation, as well as the protective effects of pravastatin. Because behavioral assessment alone cannot localize cerebral injury, and single MRI lacks direct functional validation of cognitive impairment, we combined multimodal MRI with the Morris water maze to build an integrated evaluation system linking cerebral microstructural/perfusion damage to spatial memory dysfunction—this is the core innovation of our study.

## 2 Materials and Methods

### 2.1 Rat Protocol and Experimental Design

All the experiments were performed in strict accordance with the National Institutes of Health guidelines for the use and care of animals, and the protocols used in this study were approved by the Animal Care Welfare Committee of Guizhou Medical University (2001318).

Sixty‐three female Sprague–Dawley rats aged 10–12 weeks and weighing 220–280 g. The rats were given free access to food and water. Female rats were isolated from their male counterparts except during mating. During oestrous, the SD rats were mated overnight with a female:male ratio of 1:1. In the morning, the presence of a vaginal plug was used to confirm Day 0 of pregnancy. Pregnant rats were weighed and randomly divided into three major groups: the control normal pregnant (NP) group (*n* = 21), the L‐NAME (*n* = 21) group, and the L‐NAME + pravastatin (PRA) group (*n* = 21). All 21 rats in each main group underwent blood pressure measurement at GD19. For statistical analysis and graphical presentation at GD19, seven rats were randomly selected from the 21 rats. Each group was further divided into three subgroups, namely, the 10‐day postpartum group, the 1‐month postpartum group and the 3‐month postpartum group, with seven rats in each subgroup. The normal pregnant rats in the control group received no special treatment. Before randomization into three postpartum subgroups, baseline characteristics including body weight and blood pressure were examined to ensure homogeneity and comparability among subgroups. Random allocation was performed using a computer‐generated random number table. All data at gestational Day 19 (GD19) shown in Figures 1, 2, 3 were obtained before subgroup assignment, which objectively reflected the baseline status and guaranteed balanced group composition.

**FIGURE 1:**
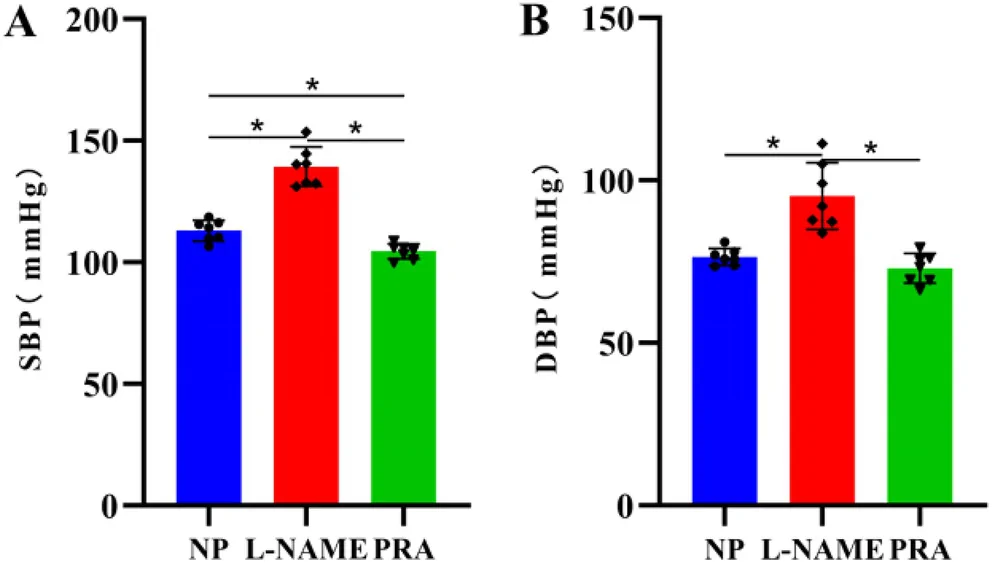
Graph showing comparison of blood pressure on GD19. (A) SBP in L‐NAME group increased significantly compared with NP group, while SBP reduced dramatically in PRA group, and was even lower than NP group. (B) DBP in L‐NAME group increased significantly compared with NP group, while DBP reduced dramatically in PRA group. Data at GD19 were obtained from seven randomly selected rats before subdivision into postpartum subgroups. Data at GD19 were obtained before subgroup randomization to ensure baseline comparability among the three postpartum subgroups. Data are shown as means ± SD (*n* = 7 rats per group). One‐way ANOVA and Bonferroni as post hoc analysis were performed to generate *p* values. *p < 0.05.

The rats in the L‐NAME group were intraperitoneally injected with L‐NAME (250 mg/kg/day) continuously on gestation Days 13–19. This dose and timing were selected based on previous studies showing reliable induction of a stable, severe PE‐like phenotype including hypertension, proteinuria, and fetal growth restriction in pregnant rats. The rats in the PRA group received the same L‐NAME regimen as the model group and were additionally administered pravastatin (5 mg/kg/day) by daily gavage on gestation Days 13–19. In this study, pravastatin was used as a mechanistic intervention to explore its protective effects against PE‐induced brain injury and cognitive dysfunction [18, 19].

### 2.2 Measurement of Blood Pressure

Caudal arterial blood pressure was measured by an animal noninvasive blood pressure apparatus (Kent Scientific) at gestational Day 19 and 10 days, 1 month, and 3 months postpartum. To minimize stress, all rats underwent 3 consecutive days of habituation training (handling, restraint, and mock measurement) before formal testing. During measurement, rats were gently restrained, and core body temperature was maintained at 37°C ± 1°C using a thermostatically controlled heating platform for 5 min to ensure stable vasodilation and reliable readings.

### 2.3 Body Weight, Incidence of Fetal Disability, and Number of Surviving Pups

The weights of the pregnant rats in each group were recorded from the 13th day to the 19th day of gestation. After delivery, the number of surviving pups, incidence of developmentally abnormal pups, and body weights of surviving pups were recorded.

Surviving pups: Pups showing spontaneous respiration and active movement within 30 min after birth.

Developmentally abnormal pups: Pups meeting any of the following criteria: Fetal growth restriction (body weight below the 10th percentile of the NP control pups). Gross morphological abnormalities (subcutaneous edema, hemorrhage, or limb deformity). Stillbirth or death within 24 h after birth.

### 2.4 Histology and Immunohistochemistry

#### 2.4.1 Urinary Albumin and Creatinine Measurements

On the 19th day of gestation, all the rats were placed in metabolic cages for 24‐h urine collection, after centrifugation, the supernatant was collected and stored at −80°C. Urinary albumin and creatinine levels were assessed in 24‐h urine samples using an albumin‐to‐creatinine ratio (ACR) assay kit (BioVision Inc., US).

#### 2.4.2 Serum Angiogenic and Inflammatory Factor Analysis

At gestational Day 19 and at 10 days, 1 month, and 3 months postpartum, approximately 3 mL of venous blood was collected from each rat via tail vein catheterization. Samples were centrifuged at 4000 rpm for 15 min at 4°C, and the supernatants were stored at −80°C. Serum levels of sFlt‐1, sEng, PlGF, IL‐6, and estradiol were measured using commercial ELISA kits. These factors are well‐recognized surrogate markers closely associated with endothelial function and systemic inflammation, reflecting vascular stress and inflammatory status. Briefly, serial dilutions of standards were prepared, and samples were added in duplicate. After incubation and washing, enzyme conjugates, substrate, and stop solution were added sequentially. Optical density was read at 450 nm, and concentrations were calculated from standard curves with appropriate dilution adjustments.

These factors are well‐recognized surrogate markers closely associated with endothelial function and systemic inflammation; they reflect vascular stress and inflammatory status and may contribute to the development of endothelial dysfunction rather than directly indicating established endothelial dysfunction [20, 21].

### 2.5 Morris Water Maze Test

The rats in each group were subjected to a water maze test at 10 days, 1 month and 3 months after delivery to evaluate spatial learning and memory.

In each trial, the rats were allowed to search for the hidden platform. The escape latency was defined as the time from entering the water to finding the platform. If a rat failed to locate the hidden platform within 90 s, the experimenter gently guided the rat to the platform and allowed it to rest on the platform for 10 s to facilitate spatial learning. For such rats, the escape latency was recorded as 90 s. The probe trial was conducted only after completion of all 4 days of training trials, not after individual missed trials. The escape latency, time spent near the former platform location and number of platform crossings were used as measures of spatial memory in the probe trial. Data from the water maze test were recorded using a video camera and Video‐Tracking System Software (Smart version 3.0.05; Panlab, Harvard).

### 2.6 MRI Protocol

All the rats were scanned with a clinical 3.0T MRI system (Discovery 750w, GEMedical System, Milwaukee, Wisconsin, USA). A 4‐channel rat coil (Jiangyin Wankang Medical Technology Co. Ltd.) was utilized. Rats were anesthetized with 4% isoflurane in oxygen for induction. Following loss of consciousness, anesthesia was maintained with 1.5%–2.0% isoflurane via a facemask with continuous oxygen delivery throughout scanning [22]. Respiratory rate was monitored continuously to maintain stable anesthesia. Total MRI scanning time was approximately 35–40 min per rat. The rats were then placed in the prone position and fixed in the coil. The MRI techniques used were coronal T2‐weighted imaging (T2WI), diffusion tensor imaging (DTI), intravoxel incoherent motion (IVIM), and dynamic contrast‐enhanced (DCE)‐MRI [4, 23, 24]. The scanning parameters were as follows:

T2WI: repetition time = 5825 ms, echo time = 122.1 ms, field of view = 40 mm × 40 mm, number of excitations = 4, slice thickness = 2 mm, no gap between slices; slices 1 4, bandwidth = 27.78 kHz.

DTI was used to evaluate white matter microstructure via fractional anisotropy (FA), which reflects microstructural integrity and axonal organization. Repetition time = 2537 ms, echo time = 91.3 ms, field of view = 50 × 50 mm, number of excitations = 16, slice thickness = 2 mm, no gap between slices; slices 11, bandwidth = 62.5 kHz, diffusion direction = 6, *b* values = 0, and 1000 s/mm^2^.

IVIM was used to assess cerebral microvascular perfusion; the pseudo‐diffusion coefficient D* reflects capillary perfusion, independent of exogenous contrast agents. Repetition time = 3317 ms, echo time = 90.4 ms, field of view = 50 × 50 mm, slice thickness = 2 mm, no gap between slices; slices 11, bandwidth = 62.5 kHz. Images were acquired at multiple *b* values (0, 10, 20, 50, 80, 100, 150, 200, 400, 800, and 1000 s/mm^2^) in the phase‐encoding direction.

DCE‐MRI was used to quantify blood–brain barrier (BBB) permeability via the transfer constant Ktrans, which is elevated when BBB integrity is disrupted. Repetition time = 7.7 ms, echo time = 2.2 ms, field of view = 80 × 80 mm, number of excitations = 1, slice thickness = 2 mm, no gap between slices; slices 14, bandwidth = 35.71 kHz; flip angle = 12°. Each period was 8 s, and there were 100 periods in total. Gadolinium‐DTPA (Gd‐DTPA) dissolved in normal saline (total volume of 1 mL) was injected manually via the tail vein within 5 s at a dose of 0.1 mmol/kg in the sixth period.

For DCE‐MRI, Gd‐DTPA (0.1 mmol/kg) was manually injected via the tail vein, a standard approach in small‐animal DCE‐MRI due to the lack of automated systems for rodents. All images were transferred to an offline workstation (Advantage Windows 4.7). Two blinded independent technicians performed ROI placement and quantitative analysis. Reproducibility was ensured by standardized ROI size/location and bilateral averaging.

DTI and DCE‐MRI images were analyzed using READY VIEW and GenIQ tools. Three 0.5 mm^2^ ROIs were drawn on the left anterior cortex, posterior cortex, and hippocampus to measure FA values, mirrored to the contralateral side and averaged. Ktrans (BBB permeability) was calculated using a modified two‐compartment model. Six 0.5 mm^2^ ROIs were placed bilaterally, and average Ktrans values were recorded. Time‐concentration and time‐signal intensity curves were also acquired.

The IVIM signal intensity relationship is expressed as follows:

$$S\left(b\right)=S\left(0\right)\left[\left(1-f\right)\cdot \exp.\left(-\mathrm{bD}\right)+f\cdot \exp.\left(-{\mathrm{bD}}^{*}\right)\right],$$

In the equation, *S*(*b*) is signal at *b* value, *S*(0) at *b* = 0, D the slow diffusion coefficient, D* the fast (pseudodiffusion) coefficient. Using ImageJ, ROIs were placed bilaterally in the anterior cortex, posterior cortex, and hippocampus (Figure 9A–C) to obtain signal intensities at different *b* values. Data were imported into MATLAB R2018b to derive D* values for the above regions.

### 2.7 Sacrifice and Organ Collection

After the MRI experiments, the rats were sacrificed by cervical dislocation under isoflurane anesthesia. Then, the brains were carefully removed and rinsed in saline. The anterior cortex, posterior cortex, and hippocampus were isolated and processed according to detection requirements: tissues for histological staining were directly fixed in 4% paraformaldehyde; tissues for molecular detection were snap‐frozen in liquid nitrogen and stored at −80°C; and tissues for transmission electron microscopy were fixed in 2.5% glutaraldehyde.

### 2.8 Hematoxylin and Eosin (HE) and Nissl Staining and Microscopy (TEM)

A Leica SCN400 Slide Scanner (Leica Microsystems, Wetzlar, Germany) was used to digitize hematoxylin and eosin (HE)‐stained and Nissl‐stained sections for quantitative histological analysis. With the use of Lecia SlidePath Gateway software, three random nonoverlapping areas of the anterior cortex, posterior cortex and hippocampus were imaged at 40× magnification. One hundred nanometer‐thick sections were stained with 2% uranyl acetate and calcinated with lead citrate and used to acquire TEM images with an HT7700 (Hitachi) transmission electron microscope.

### 2.9 Statistical Analysis

The primary outcomes of the MRI analysis were the between‐group comparisons of FA, D*, and Ktrans values at each postpartum time point, as well as their correlations with escape latency. Secondary outcomes included the longitudinal changes within each group. All comparisons were prespecified.

All statistical analyses were performed using GraphPad Prism 9.0 software, data are expressed as mean ± standard deviation (SD) for normally distributed data or median (interquartile range, IQR) for non‐parametric data, the Shapiro–Wilk test was used to evaluate normality and Levene's test was used to assess homogeneity of variance, one‐way analysis of variance (ANOVA) followed by Bonferroni post‐hoc test was used for comparisons among three or more groups at a single time point, two‐way ANOVA followed by Bonferroni post‐hoc test was used for comparisons involving group × time interactions (longitudinal data), Kruskal–Wallis *H* test was used for non‐normally distributed data, Pearson correlation analysis was performed for normally distributed continuous variables and Spearman correlation analysis was used for non‐parametric variables, a significance level of *p* < 0.05 was considered statistically significant and the sample size was *n* = 7 rats per subgroup.

## 3 Results

### 3.1 PE Model Establishment, Fetal Outcomes, and Proteinuria

L‐NAME administration successfully induced typical PE‐like phenotypes in pregnant rats (Figures 1, 2, 3). On GD19, systolic and diastolic blood pressures were significantly elevated in the L‐NAME group compared with the NP group (both *p* < 0.0001), and were markedly reduced by pravastatin (both *p* < 0.0001; Figure 1). The L‐NAME group also exhibited lower maternal weight gain, fewer surviving pups, lower fetal weights, and higher fetal disability rates (all *p* < 0.01; Figure 2). Pravastatin significantly improved fetal survival and growth (*p* < 0.05). In addition, 24‐h urinary albumin and creatinine levels were markedly increased in the L‐NAME group (*p* < 0.0001) and were significantly alleviated by pravastatin (*p* < 0.0001; Figure 3).

**FIGURE 2:**
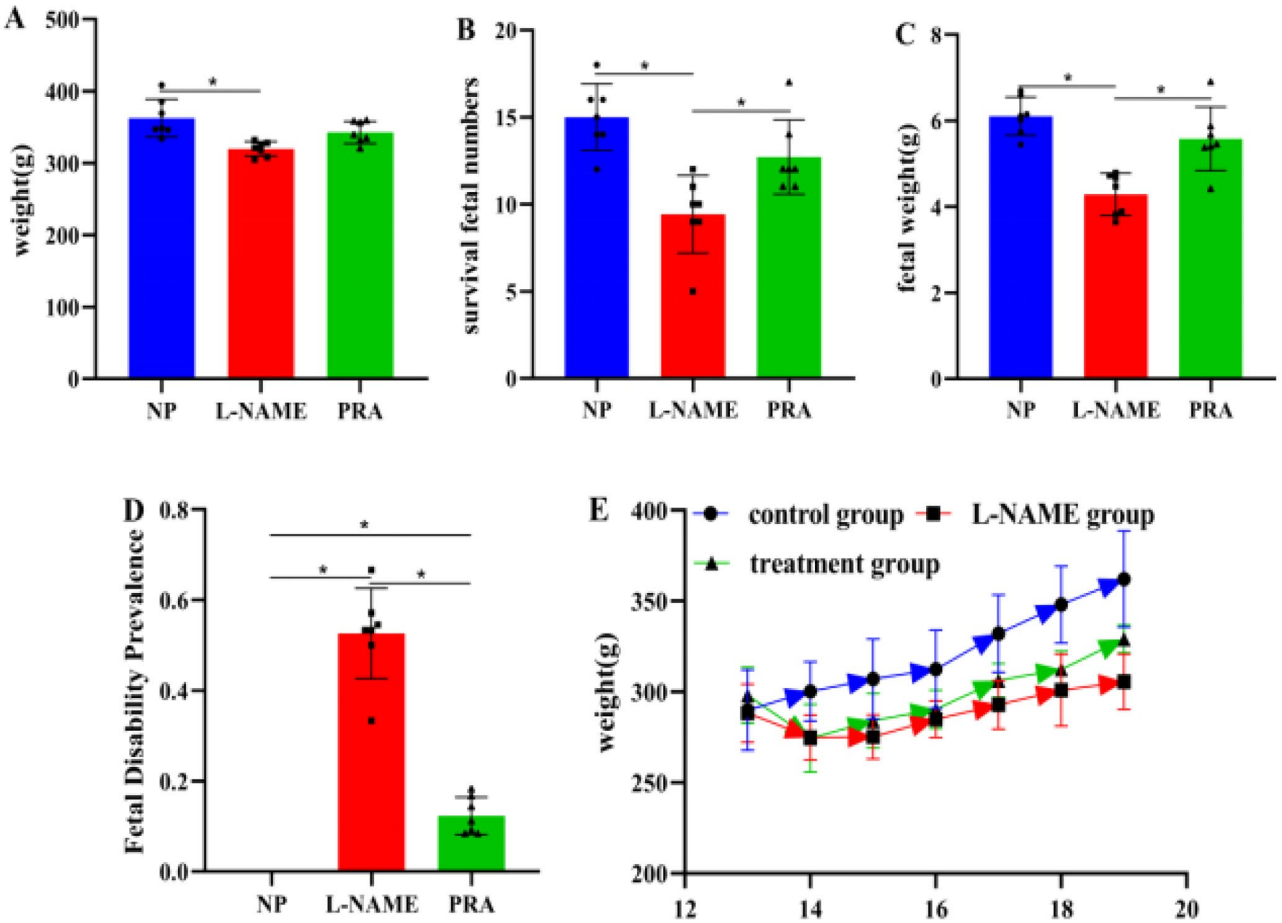
Comparison of the weight of pregnant rats and the number of pups and the weight difference of the pups. (A) The weight of pregnant rats in the L‐NAME group was significantly lighter than that of the NP group, while the weight gained in the PRA group. (B) The number of surviving pups at birth in the L‐NAME group was significantly less than the NP group, while the number increased remarkably in the PRA group. (C) The fetal weight in the L‐NAME group reduced significantly compared to the NP group, while the fetal weight increased dramatically in the PRA group. (D) The pups in the L‐NAME group presented higher rates of disability, which improved significantly by pravastatin treatment in the PRA group. However, the difference between the NP group and the PRA group still existed. (E) The range of body weight on GD13‐19 in the L‐NAME group (the red line) was lower than that in the NP group (the blue line), while the range in the PRA group (the green line) was higher than that in the L‐NAME group, but remained lower than NP group. Data at GD19 were obtained before subgroup randomization to ensure baseline comparability among the three postpartum subgroups. Data are shown as means ± SD (*n* = 7 rats per group). One‐way ANOVA and Bonferroni as post hoc analysis were performed to generate *p* values. *p < 0.05.

**FIGURE 3:**
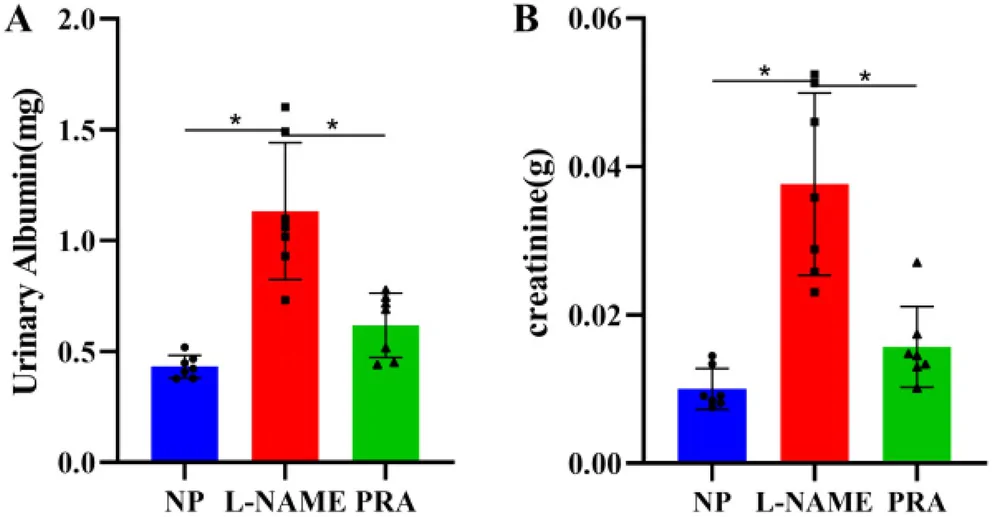
Graph showing comparison of the total amount of 24 h urinary albumin and creatinine on GD19. (A) Urinary albumin of 24 h in the L‐NAME group increased significantly compared with the NP group, while urinary albumin in the PRA group decreased dramatically. (B) Urinary creatinine in the L‐NAME group increased significantly compared with NP group, which decreased dramatically in the PRA group. Data at GD19 were obtained before subgroup randomization to ensure baseline comparability among the three postpartum subgroups. Data are shown as means ± SD (*n* = 7 rats per group). One‐way ANOVA and Bonferroni as post hoc analysis were performed to generate *p* values. *p < 0.05.

### 3.2 Serum Markers of Endothelial Dysfunction and Inflammation

The L‐NAME group exhibited obvious angiogenic imbalance and systemic inflammation (Figure 4). Serum sEng and IL‐6 levels were significantly higher in the L‐NAME group than in the NP group (both *p* < 0.0001) and were dramatically decreased by pravastatin (both *p* < 0.0001; Figure 4A,C). PlGF levels were significantly lower in the L‐NAME group (*p* < 0.0001), partially restored by pravastatin (*p* < 0.05), but still lower than in the NP group (*p* < 0.0001; Figure 4B). These results indicate that L‐NAME induces endothelial injury and inflammation, which are effectively ameliorated by pravastatin.

**FIGURE 4:**
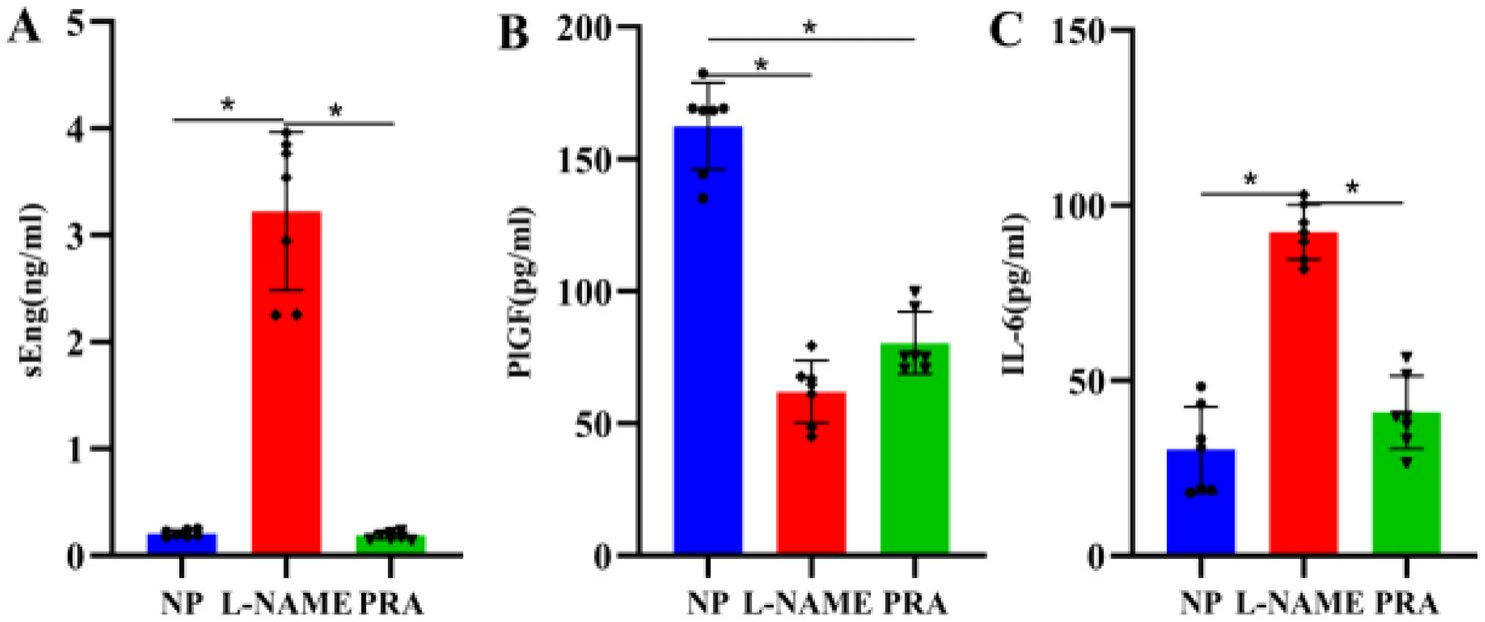
Graph showing comparison of circulating factors in serum on GD19. (A) sEng in L‐NAME group increased significantly compared with the NP group, which reduced dramatically in the PRA group. (B) PlGF in the L‐NAME group decreased significantly compared with the NP group, which increased slightly in the PRA group, but remained significantly lower than the NP group. (C) IL‐6 in L‐NAME group increased significantly compared with the NP group, which reduced dramatically in the PRA group. Data at GD19 were obtained before subgroup randomization to ensure baseline comparability among the three postpartum subgroups. Data are shown as means ± SD (*n* = 7 rats per group). One‐way ANOVA and Bonferroni as post hoc analysis were performed to generate *p* values. *p < 0.05.

### 3.3 Cognitive Function Impairment in PE Rats

PE rats exhibited significant and persistent spatial learning and memory deficits (Figures 5 and 6). The L‐NAME group showed markedly longer escape latency, fewer platform crossings, and less time spent in the target quadrant than the NP group (all *p* < 0.0001). These cognitive deficits remained stable across all three postpartum time points. Pravastatin treatment significantly improved all cognitive indices (all *p* < 0.01), with no significant difference compared with the NP group. Behavioral tracking confirmed disorganized searching patterns in PE rats, which were partially normalized by pravastatin (Figure 6).

**FIGURE 5:**
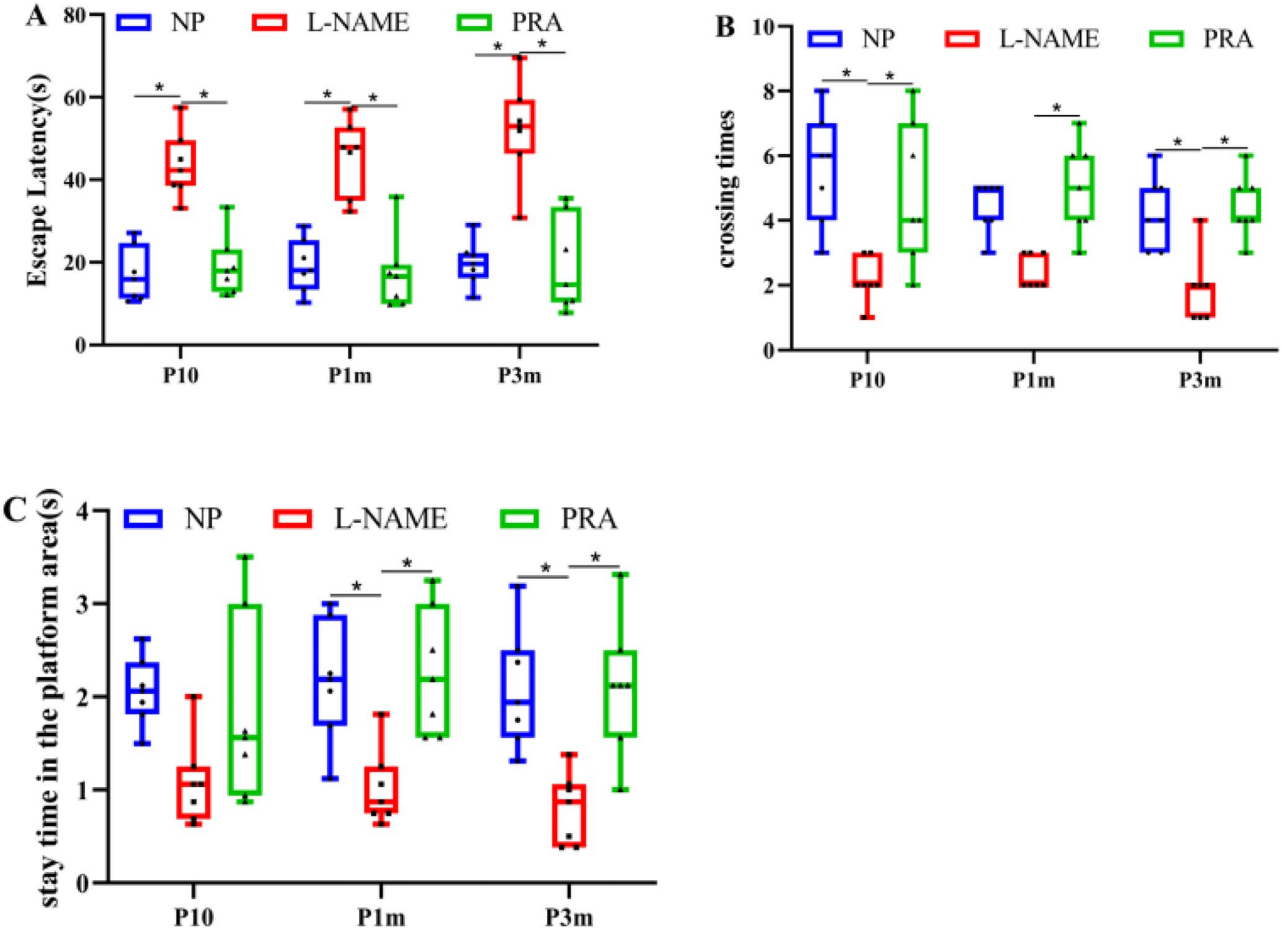
Spatial memory was impaired in the L‐NAME group in the probe trial at 10 days, 1 month and 3 months postpartum. (A) Latency to escape onto the training platform area of the Morris water maze at different time points. Escape latency in the L‐NAME group was significantly longer than NP group at three time points, which was dramatically shorter in the PRA group. The difference between the NP group and the PRA group was not significant. The escape latency of the L‐NAME group prolonged over time. (B) Frequency of crossing over the platform area at different time points. Crossing times in the L‐NAME group was dramatically less than the NP group at three time points, which increased significantly in the PRA group. The difference between the NP group and the PRA group was not significant. The crossing times of the L‐NAME group decreased over time. (C) Stay time in the platform area at different time points. Stay time in the L‐NAME group was dramatically less than the NP group at three time points, which increased notably in the PRA group. The difference between the NP group and the PRA group was not significant. The stay time of the L‐NAME group reduced over time. Data are shown as means ± SD (*n* = 7 rats per group). Two‐way ANOVA and Bonferroni as post hoc analysis were performed to generate *p* values. *p < 0.05. There was no significant interaction between time and grouping factors in escape latency, crossing times and stay time in the platform area.

**FIGURE 6:**
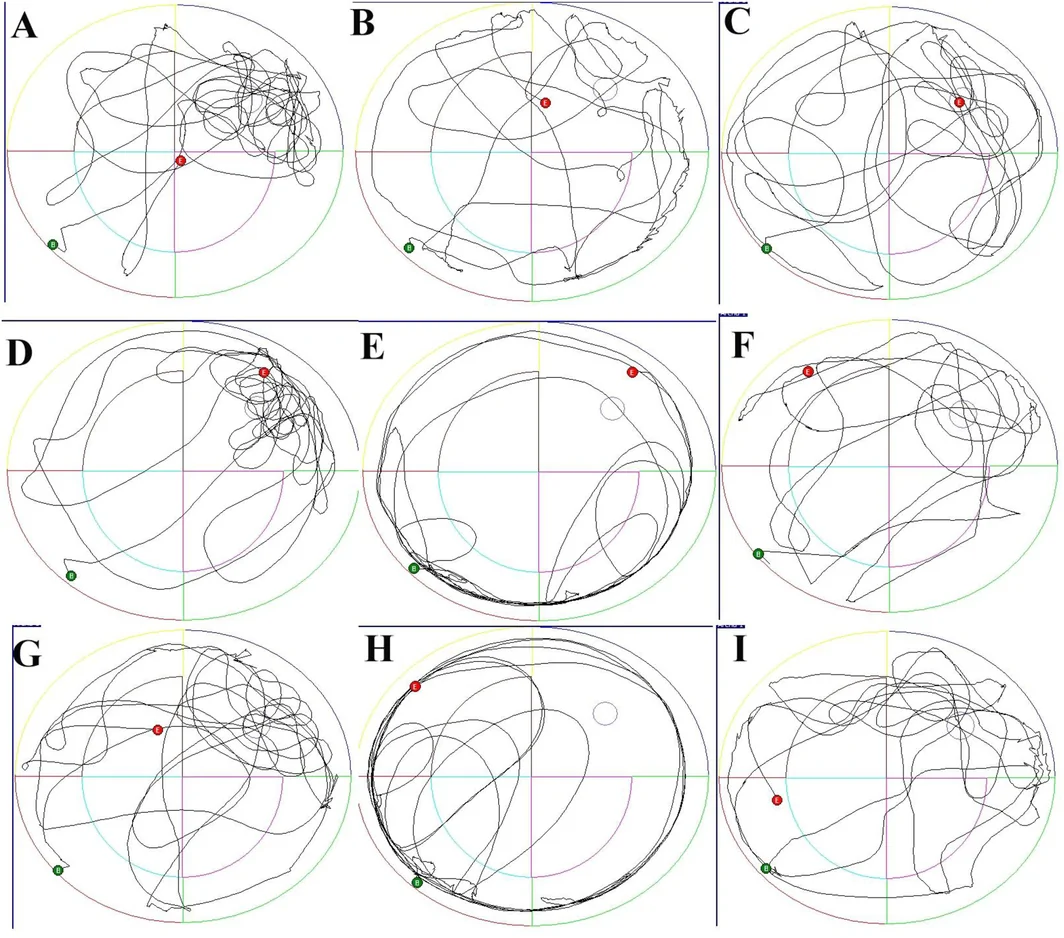
Typical behavior tracks of the tested rats in the probe trial at 10 days, 1 month and 3 months postpartum. (A) Tracks of rats in the NP group at 10 days postpartum. (B) Tracks of rats in the L‐NAME group at 10 days postpartum. (C) Tracks of rats in the PRA group at 10 days postpartum. (D) Tracks of rats in the NP group at 1 month postpartum. (E) Tracks of rats in the L‐NAME group at 1 month postpartum. (F) Tracks of rats in the PRA group at 1 month postpartum. (G) Tracks of rats in the NP group at 3 months postpartum. (H) Tracks of rats in the L‐NAME group at 3 months postpartum. (I) Tracks of rats in the PRA group at 3 months postpartum.

### 3.4 Multimodal MRI Assessment of Brain Injury

To systematically evaluate the neural substrates of PE‐induced cognitive impairment, we examined three complementary MRI parameters: FA, D*, and Ktrans.

#### 3.4.1 FA Values

FA values reflect white matter microstructural integrity. FA maps visually demonstrated reduced white matter integrity in the L‐NAME group, which was improved by pravastatin (Figure 7). Two‐way ANOVA revealed significant main effects of group and time (all *p* < 0.0001) in the anterior cortex, posterior cortex, and hippocampus (Figure 8A–C).

**FIGURE 7:**
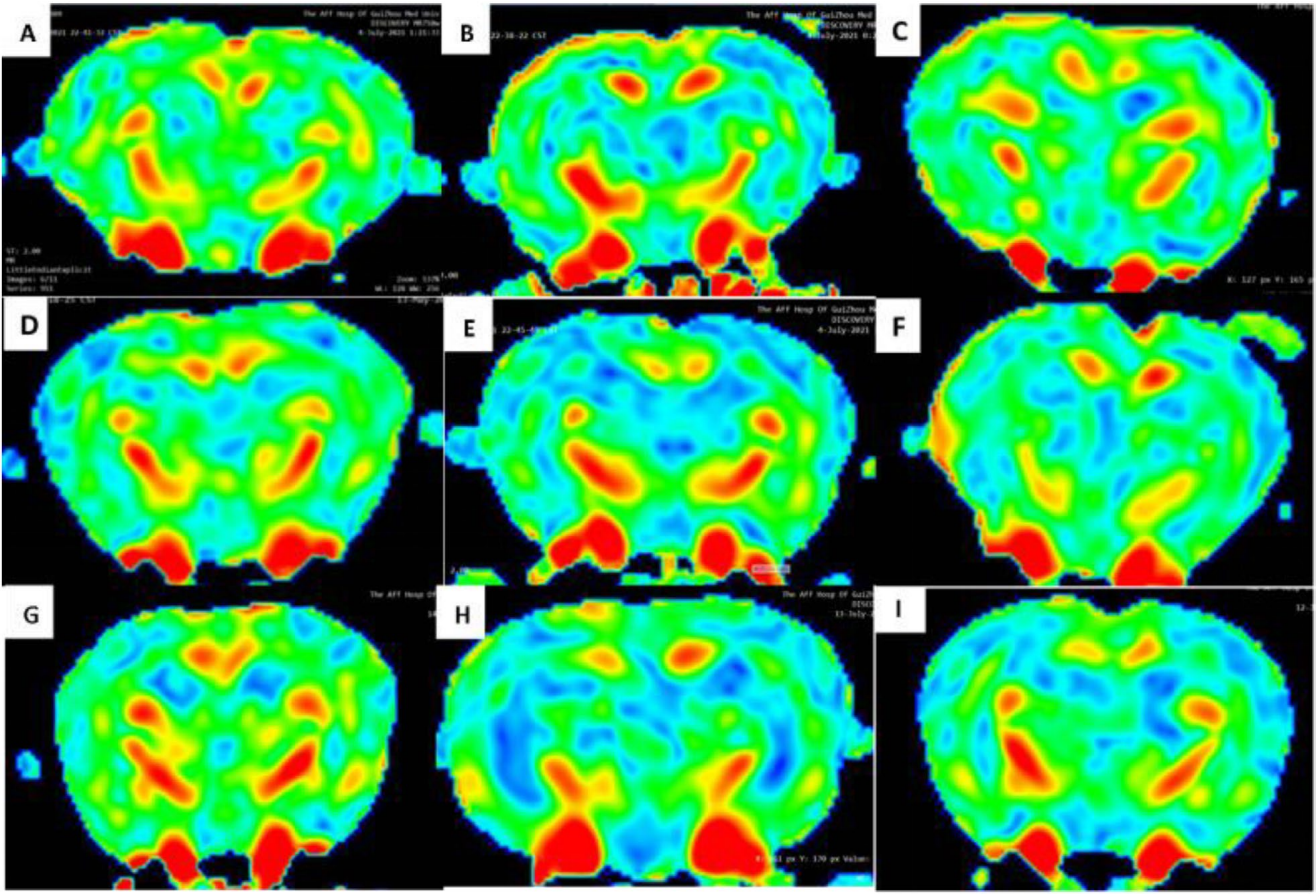
Comparison of FA maps over time. FA values range from 0 to 1. The color closer to blue means the FA values are closer to 0, while the color closer to red means closer to 1. (A) FA maps of rats in the NP group at 10 days postpartum. (B) FA maps of rats in the L‐NAME group at 10 days postpartum. (C) FA maps of rats in the PRA group at 10 days postpartum. (D) FA maps of rats in the NP group at 1 month postpartum. (E) FA maps of rats in the L‐NAME group at 1 month postpartum. (F) FA maps of rats in the PRA group at 1 month postpartum. (G) FA maps of rats in the NP group at 3 months postpartum. (H) FA maps of rats in the L‐NAME group at 3 months postpartum. (I) FA maps of rats in the PRA group at 3 months postpartum.

**FIGURE 8:**
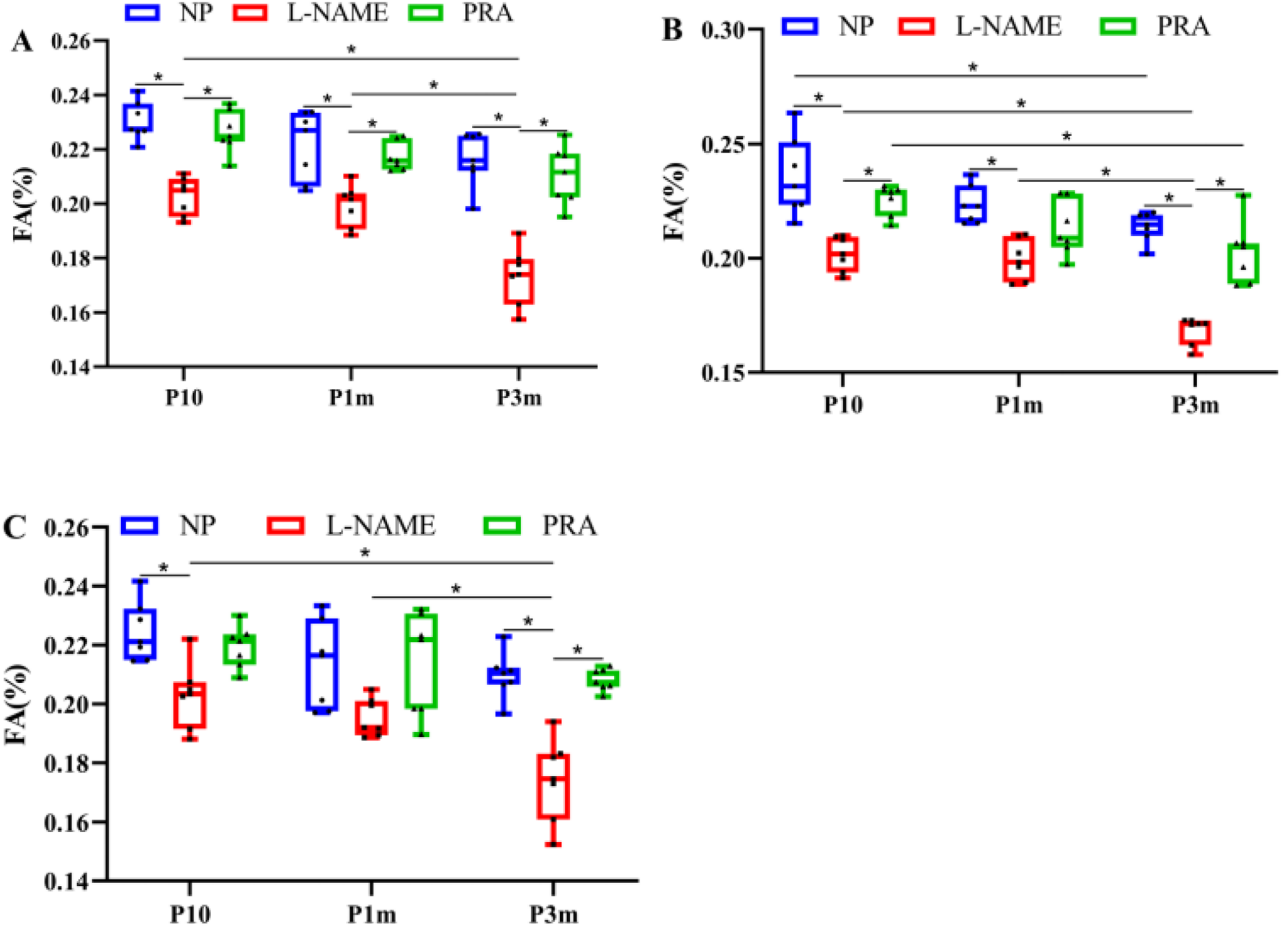
Comparison of FA values in the anterior cortex, posterior cortex and hippocampus over time. (A) FA values in the anterior cortex at different time points. FA values in the L‐NAME group were significantly lower than the NP group at three time points, which increased significantly in the PRA group. The difference between the NP group and the PRA group was not significant. The FA values decreased with time in three groups. (B) FA values in the posterior cortex at different time points. FA values in the L‐NAME group were significantly lower than the NP group at three time points, which increased dramatically in the PRA group. The difference between the NP group and the PRA group was not significant. The FA values decreased with time in three groups. (C) FA values in the hippocampus at different time points. FA values in the L‐NAME group were remarkably lower than the NP group at three time points, which increased dramatically in the PRA group. The difference between the NP group and the PRA group was not significant. FA values decreased with time in three groups as a whole. Data are shown as means ± SD (*n* = 7 rats per group). Two‐way ANOVA and Bonferroni as post hoc analysis were performed to generate *p* values. *p < 0.05. There was a significant difference in both time and grouping factors in three areas, but there was no significant interaction between time and grouping factors except in anterior cortex.

FA values were significantly lower in the L‐NAME group than in the NP group at all time points (all *p* < 0.001) and declined progressively over time, with the most severe reduction in the L‐NAME group (*p* < 0.0001). Pravastatin significantly increased FA values compared with the L‐NAME group (*p* < 0.01; Figure 8A–C), indicating preserved white matter microstructure.

Having established that PE impairs white matter microstructure, we next asked whether cerebral microvascular perfusion is also compromised.

#### 3.4.2 *D* * Values

D* represents cerebral microvascular perfusion. Two‐way ANOVA showed that D* values were significantly affected by group (all *p* < 0.0001) but not by time or group–time interaction (all *p* > 0.05; Figure 9A–C).

**FIGURE 9:**
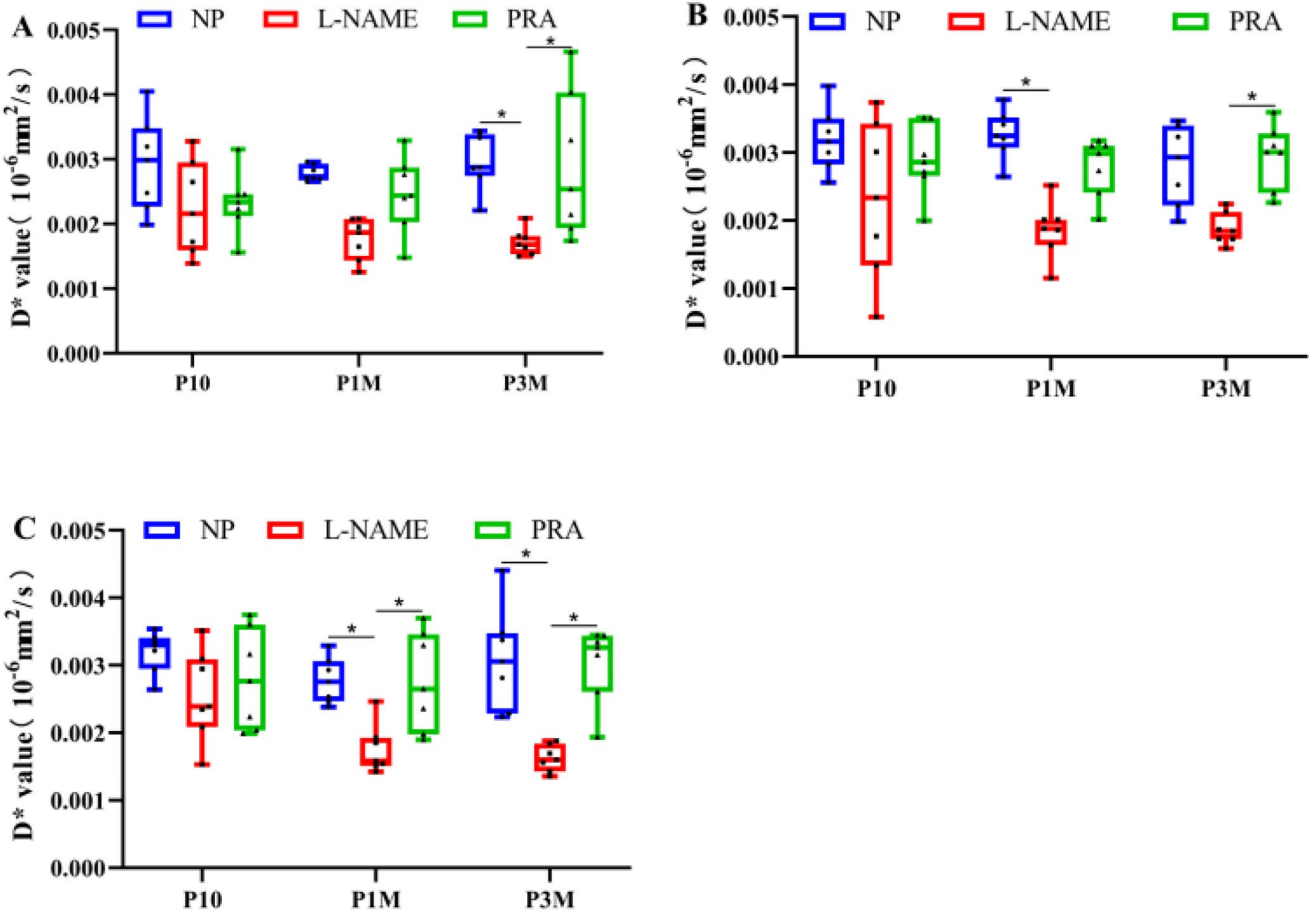
Comparison of D* values in the anterior cortex, posterior cortex, and hippocampus over time. (A) D* values in the anterior cortex at different time points. D* values in the L‐NAME group were notably lower than the NP group, which increased in the PRA group except at 10 days postnatal. The difference between the NP and the PRA group was not significant. D* values decreased with time in the L‐NAME group. (B) D* values in the posterior cortex at different time points. D* values in the L‐NAME group were lower than the NP group at three time points, which increased in the PRA group. The difference between the NP and the PRA group was not significant. D* values decreased with time in three groups in the mass. (C) D* values in hippocampus at different time points. D* values in the L‐NAME group were lower than the NP group at three time points, which increased dramatically in the PRA group. The difference between the NP and the PRA group was not significant. D* values decreased remarkably with time in the L‐NAME group. Data are shown as means ± SD (*n* = 7 rats per group). Two‐way ANOVA and Bonferroni as post hoc analysis were performed to generate *p* values. *p < 0.05. There was a significant difference in grouping factors rather than time in three areas, but there was no significant interaction between time and grouping factors.

D* values were significantly lower in the L‐NAME group than in the NP group across all three brain regions and all time points, indicating markedly reduced cerebral perfusion in PE rats. Pravastatin treatment significantly restored D* values (all *p* < 0.05; Figure 9A–C), indicating improved microvascular perfusion.

#### 3.4.3 Ktrans Values

Ktrans directly reflects blood–brain barrier (BBB) permeability. Typical DCE‐MRI kinetic curves and parametric maps are shown in Figure 10. Two‐way ANOVA revealed a significant main effect of group (all *p* < 0.0001) but no effect of time or interaction (all *p* > 0.05; Figure 11A–C).

**FIGURE 10:**
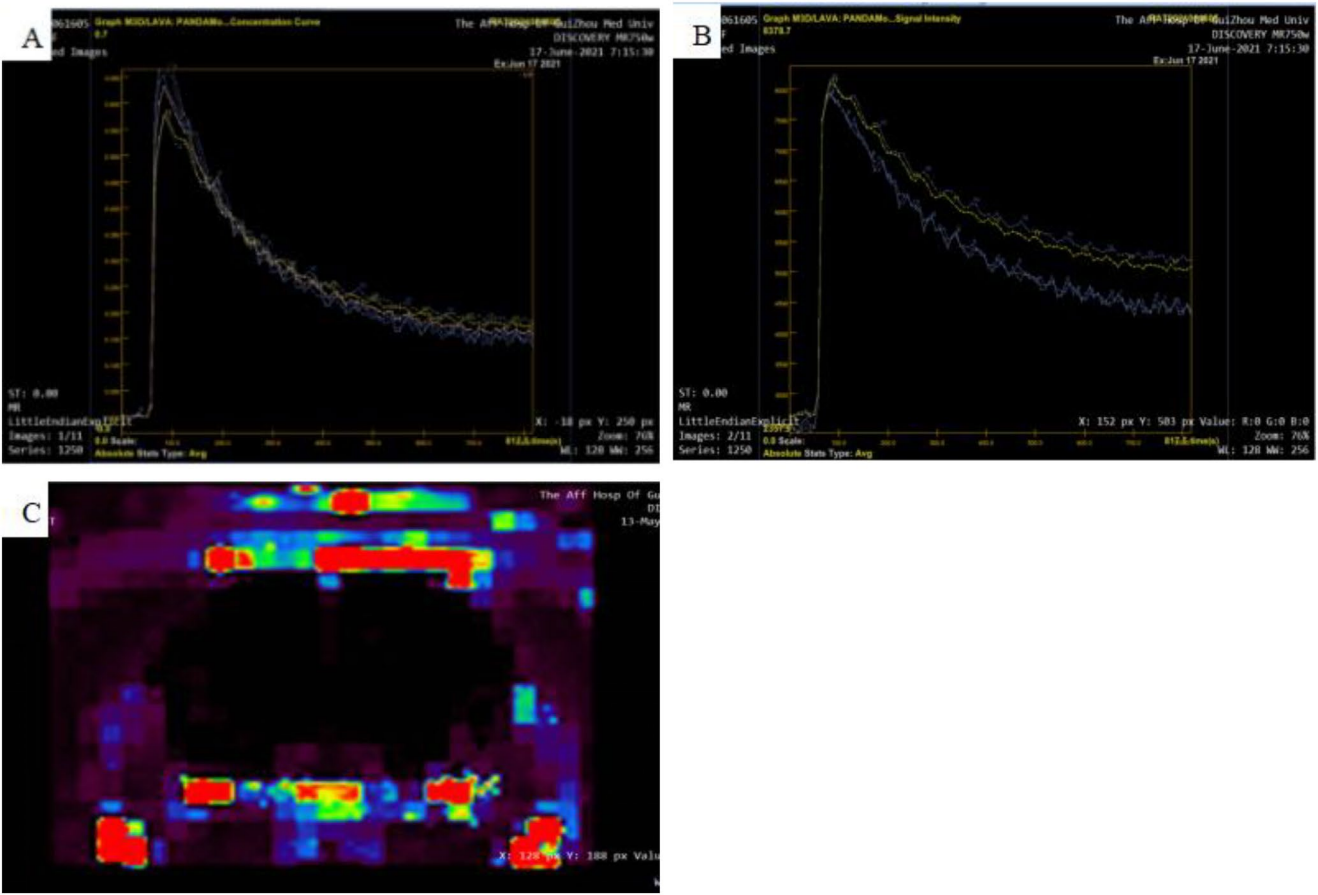
Time‐concentration curve (A), time‐signal intensity curve (B), and Ktrans map (C) of a rat produced by the DCE sequence.

**FIGURE 11:**
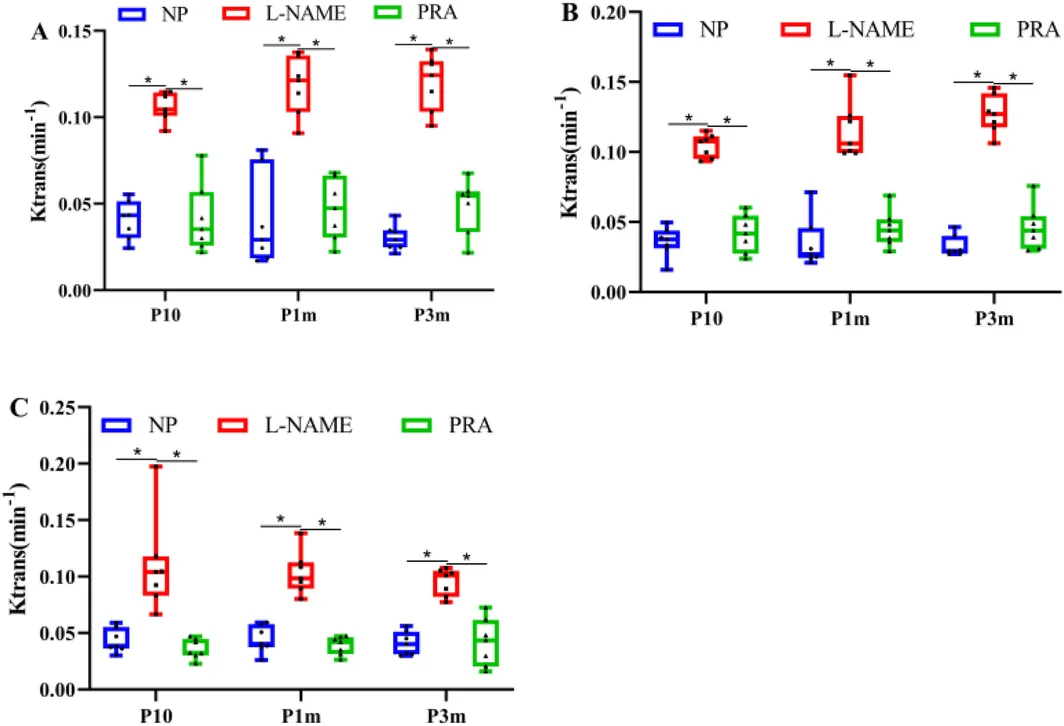
Comparison of Ktrans in the anterior cortex, posterior cortex, and hippocampus over time. (A) Ktrans in the anterior cortex at different time points. Ktrans in the L‐NAME group increased significantly compared to the NP group at three time points, which decreased significantly in the PRA group. The difference between the NP group and the PRA group was not significant. Ktrans increased with time in the L‐NAME group. (B) Ktrans in the posterior cortex at different time points. Ktrans in the L‐NAME group increased significantly compared to the NP group at three time points, which decreased significantly in the PRA group. The difference between the NP group and the PRA group was not significant. Ktrans increased over time in the L‐NAME group. (C) Ktrans in hippocampus at different time points. Ktrans in the L‐NAME group increased significantly compared to the NP group at three time points, which decreased significantly in the PRA group. The difference between the NP group and the PRA group was not significant. Data are shown as means ± SD (*n* = 7 rats per group). Two‐way ANOVA and Bonferroni as post hoc analysis were performed to generate *p* values. *p < 0.05. There was a significant difference in grouping factors in three areas, but there was no significant interaction between time and grouping factors.

Ktrans values were drastically elevated in the anterior cortex, posterior cortex, and hippocampus in the L‐NAME group relative to the NP group at all time points (all *p* < 0.0001), indicating severe BBB disruption. Pravastatin significantly reduced Ktrans values in all regions (all *p* < 0.0001; Figure 11A–C), indicating restored BBB integrity.

### 3.5 Correlations Between MRI Parameters and Cognitive Function

Pearson correlation analysis was performed between escape latency and MRI parameters in the anterior cortex, posterior cortex, and hippocampus (Tables 1, 2, 3). FA and D* values were negatively correlated with escape latency, whereas Ktrans values were positively correlated with escape latency in all three regions (all *p* < 0.0001). The *f* value showed no significant correlation with cognitive performance. These results confirm that multimodal MRI parameters sensitively reflect the severity of postpartum cognitive dysfunction in PE rats.

**TABLE 1:** Correlation between escape latency and MRI parameters in anterior cortex.

MRI parameter	*r*	*R* ^2^	95% CI	*p*
FA	−0.6789	0.4609	(−0.7932, −0.5183)	< 0.0001
D*	−0.5261	0.2768	(−0.6856, −0.3200)	< 0.0001
Ktrans	0.7837	0.6142	(0.6651, 0.8638)	< 0.0001

*Note:* FA and D* values were negatively correlated with escape latency, while Ktrans values were positively correlated with escape latency in anterior cortex. Statistics: Pearson Correlation.

Abbreviations: CI, confidence interval; *r*, correlation coefficient; *R* ^2^, coefficient of determination.

**TABLE 2:** Correlation between escape latency and MRI parameters in posterior cortex.

MRI parameter	*r*	*R* ^2^	95% CI	*p*
FA	−0.6580	0.4330	(−0.7788, −0.4902)	< 0.0001
D*	−0.5449	0.2969	(−0.6984, −0.3435)	< 0.0001
Ktrans	0.8390	0.7038	(0.7463, 0.8997)	< 0.0001

*Note:* FA and D* values were negatively correlated with escape latency, while Ktrans values were positively correlated with escape latency in posterior cortex. Statistics: Pearson Correlation.

Abbreviations: CI, confidence interval; *r*, correlation coefficient; *R* ^2^, coefficient of determination.

**TABLE 3:** Correlation between escape latency and MRI parameters in hippocampus.

MRI parameter	*r*	*R* ^2^	95% CI	*p*
FA	−0.6422	0.4124	(−0.7678, −0.4690)	< 0.0001
D*	−0.5109	0.2610	(−0.6734, −0.3013)	< 0.0001
Ktrans	0.7169	0.5139	(0.5704, 0.8192)	< 0.0001

*Note:* FA and D* values were negatively correlated with escape latency, while Ktrans values were positively correlated with escape latency in hippocampus. Statistics: Pearson Correlation.

Abbreviations: CI, confidence interval; *r*, correlation coefficient; *R* ^2^, coefficient of determination.

### 3.6 Hippocampal Neuronal Injury Detected by HE and Nissl Staining

To validate whether the MRI‐detected microstructural and perfusion abnormalities correspond to actual neuronal damage, we performed histological analysis of the hippocampus—a key region for learning and memory. Histological staining revealed progressive hippocampal neuronal injury in PE rats (Figures 12 and 13). Compared with the NP group, the L‐NAME group exhibited obvious neuronal degeneration, nuclear pyknosis, disorganized arrangement, and thinning of the neuronal layer, which gradually deteriorated over 3 months. Pravastatin treatment significantly alleviated neuronal damage and restored neuronal morphology and structural arrangement.

**FIGURE 12:**
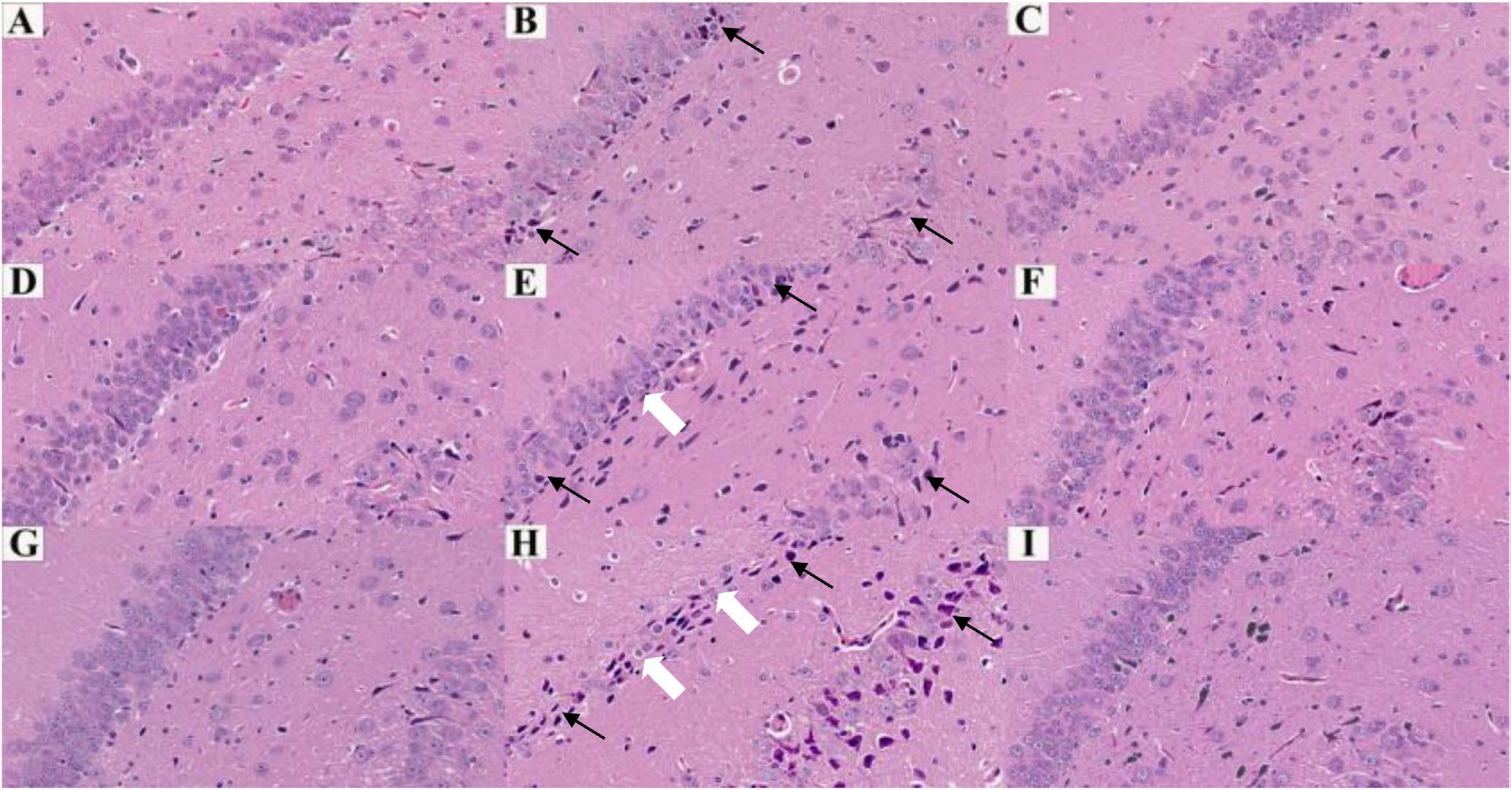
Comparison of HE staining in the hippocampus over time (×200). The black arrows showed abnormal morphology of neurons with hyperchromatic nuclei, while the white arrows showed a sparse arrangement of neurons with a significantly reduced number compared with the control group. (A) HE staining of rat in the NP group at 10 days postpartum. (B) HE staining of rat in the L‐NAME group at 10 days postpartum. (C) HE staining of rat in the PRA group at 10 days postpartum. (D) HE staining of rat in the NP group at 1 month postpartum. (E) HE staining of rat in the L‐NAME group at 1 month postpartum. (F) HE staining of rat in the PRA group at 1 month postpartum. (G) HE staining of rat in the NP group at 3 months postpartum. (H) HE staining of rat in the L‐NAME group at 3 months postpartum. (I) HE staining of rat in the PRA group at 3 months postpartum.

**FIGURE 13:**
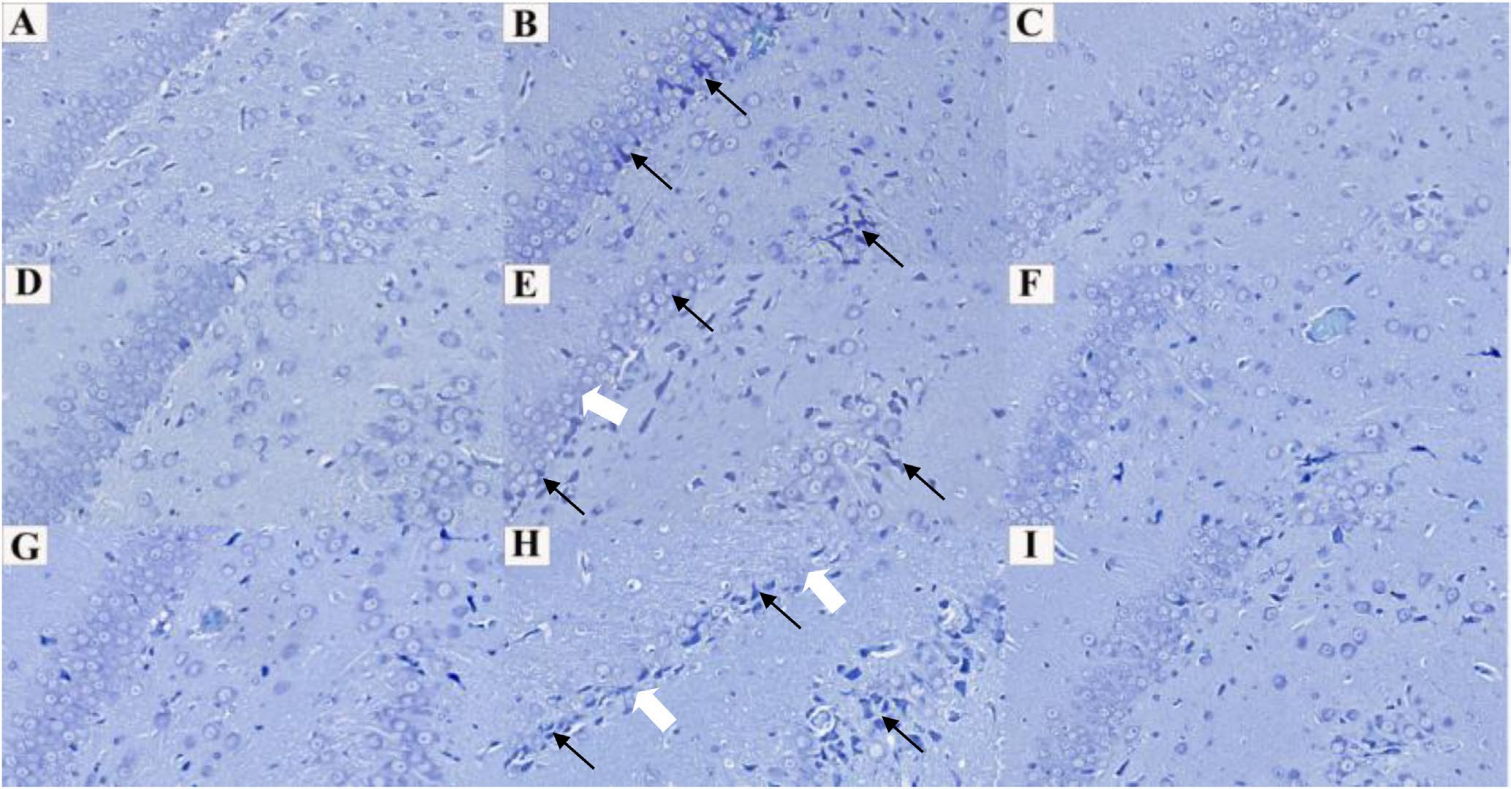
Comparison of Nissl staining in the hippocampus over time (×200). The black arrows showed abnormal morphology of neurons with hyperchromatic nuclei, while the white arrows showed a sparse arrangement of neurons with a significantly reduced number compared with the control group. (A) Nissl staining of rat in the NP group at 10 days postpartum. (B) Nissl staining of rat in the L‐NAME group at 10 days postpartum. (C) Nissl staining of rat in the PRA group at 10 days postpartum. (D) Nissl staining of rat in the NP group at 1 month postpartum. (E) Nissl staining of rat in the L‐NAME group at 1 month postpartum. (F) Nissl staining of rat in the PRA group at 1 month postpartum. (G) Nissl staining of rat in the NP group at 3 months postpartum. (H) Nissl staining of rat in the L‐NAME group at 3 months postpartum. (I) Nissl staining of rat in the PRA group at 3 months postpartum.

### 3.7 Progressive Ultrastructural Brain Injury Observed by TEM

To further confirm BBB disruption at the ultrastructural level, we performed transmission electron microscopy. Transmission electron microscopy (TEM) was used to observe ultrastructural changes, with clear labels for key structures including the blood–brain barrier (BBB), mitochondria (Mit), endothelial cells, and astrocyte foot processes (Figures 14, 15, 16).

**FIGURE 14:**
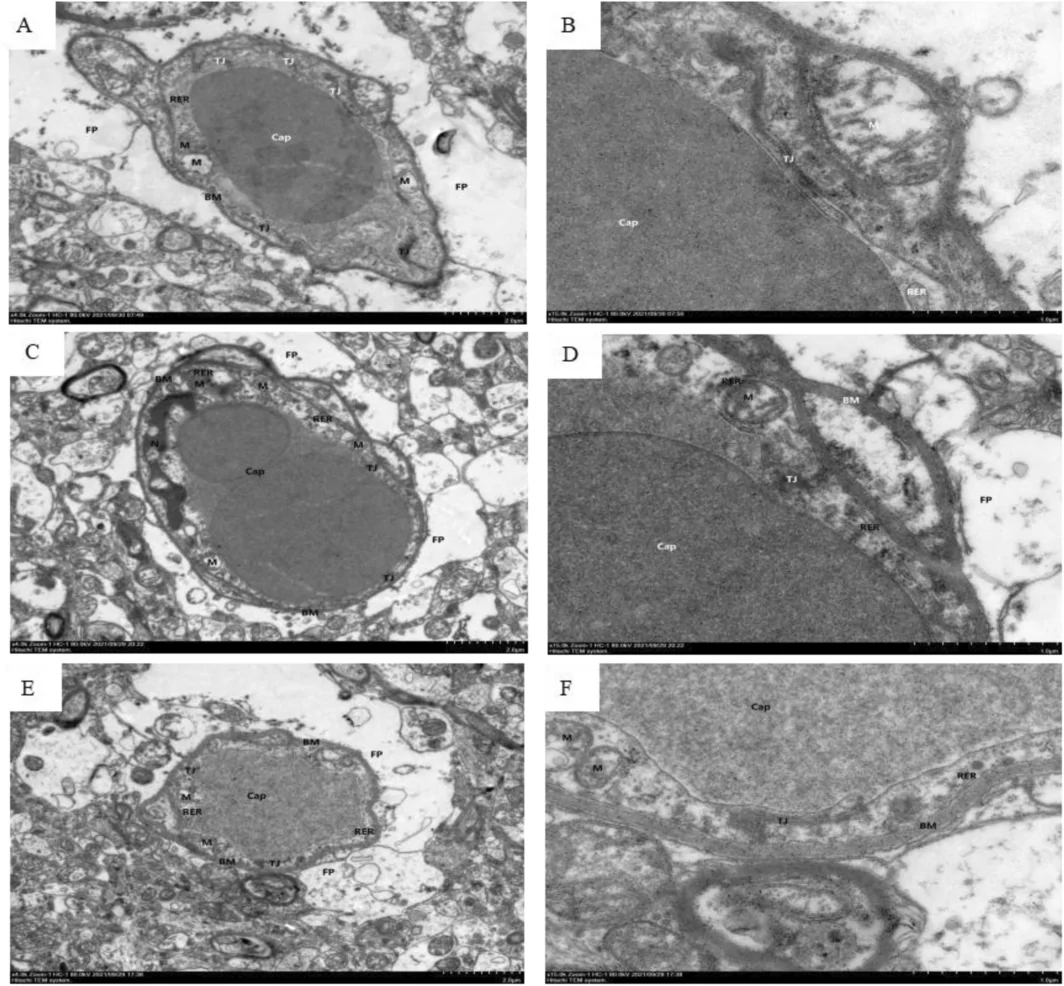
Representative electron microscopic images of rats in the L‐NAME group in the anterior cortex over time (A, C, E ×500; B, D, F ×1000). (A) TEM of rat in L‐NAME group at 10 days postpartum (×500). (B) TEM of rat in L‐NAME group at 10 days postpartum (×1000). (C) TEM of rat in L‐NAME group at 1 month postpartum (×500). (D) TEM of rat in L‐NAME group at 1 month postpartum (×1000). (E) TEM of rat in L‐NAME group at 3 months postpartum (×500). (F) TEM of rat in L‐NAME group at 3 months postpartum (×1000). AFP, astrocyte foot process; BBB, blood–brain barrier; Cap, capillary lumen; EC, endothelial cell; Mit, mitochondria; RER, rough endoplasmic reticulum. Black arrows indicate tight junction disruption; red arrows indicate swollen mitochondria; yellow arrows indicate damaged basement membrane.

TEM demonstrated gradually exacerbated BBB damage and cellular ultrastructural lesions in the anterior cortex, posterior cortex, and hippocampus of PE rats across 10 days, 1 month, and 3 months postpartum (Figures 14, 15, 16).

In the anterior cortex, mild BBB impairment, mitochondrial swelling, and astrocytic footplate edema appeared at 10 days and progressively worsened at 1 and 3 months (Figure 14A–F). In the posterior cortex, moderate BBB damage, capillary lumen stenosis, and mitochondrial injury were observed at 10 days and deteriorated over time (Figure 15A–F). In the hippocampus, mild BBB disruption and mitochondrial damage occurred at 10 days and developed into severe mitochondrial vacuolation and marked BBB disruption at 3 months (Figure 16A–F).

**FIGURE 15:**
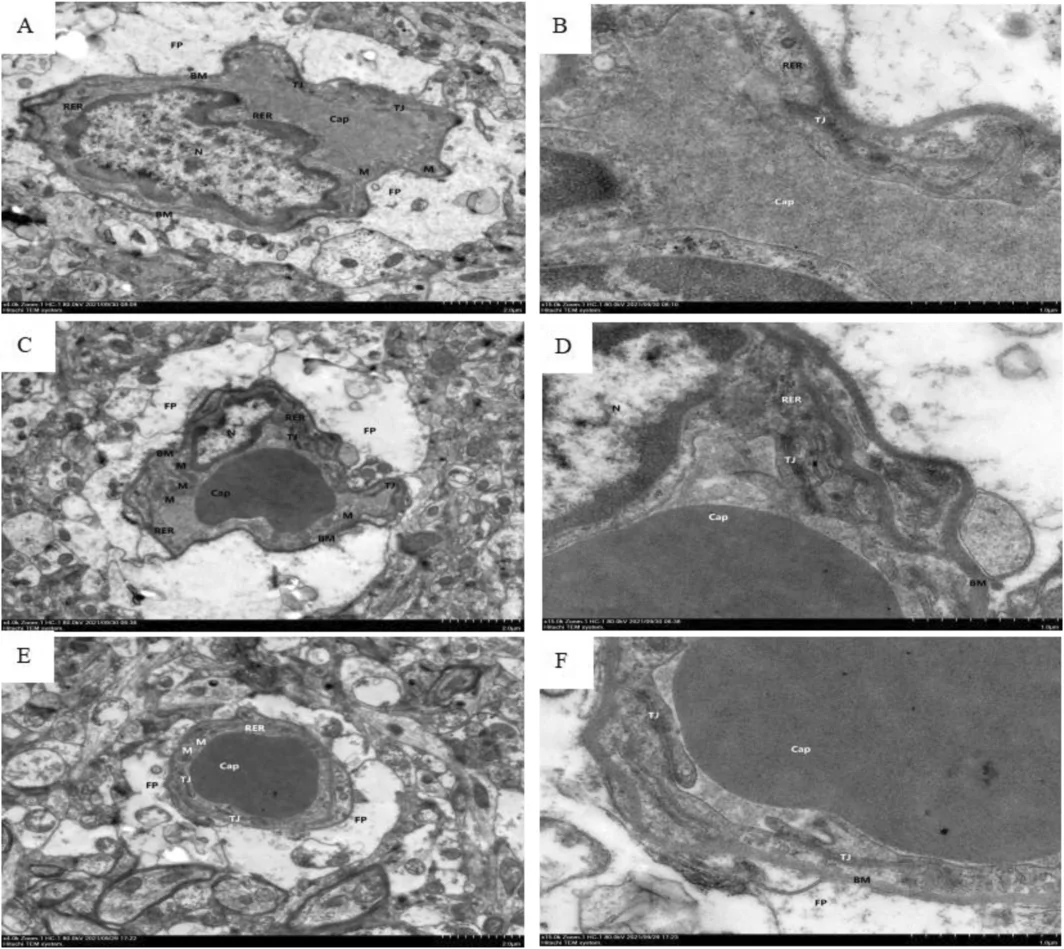
Comparison of TEM in the posterior cortex of the PE group over time (A, C, E ×500; B, D, F ×1000). (A) TEM of rat in L‐NAME group at 10 days postpartum (×500). (B) TEM of rat in L‐NAME group at 10 days postpartum (×1000). (C) TEM of rat in L‐NAME group at 1 month postpartum (×500). (D) TEM of rat in L‐NAME group at 1 month postpartum (×1000). (E) TEM of rat in L‐NAME group at 3 months postpartum (×500). (F) TEM of rat in L‐NAME group at 3 months postpartum (×1000). AFP, astrocyte foot process; BBB, blood–brain barrier; Cap, capillary lumen; EC, endothelial cell; Mit, mitochondria; RER, rough endoplasmic reticulum. Black arrows indicate tight junction disruption; red arrows indicate swollen mitochondria; yellow arrows indicate damaged basement membrane.

**FIGURE 16:**
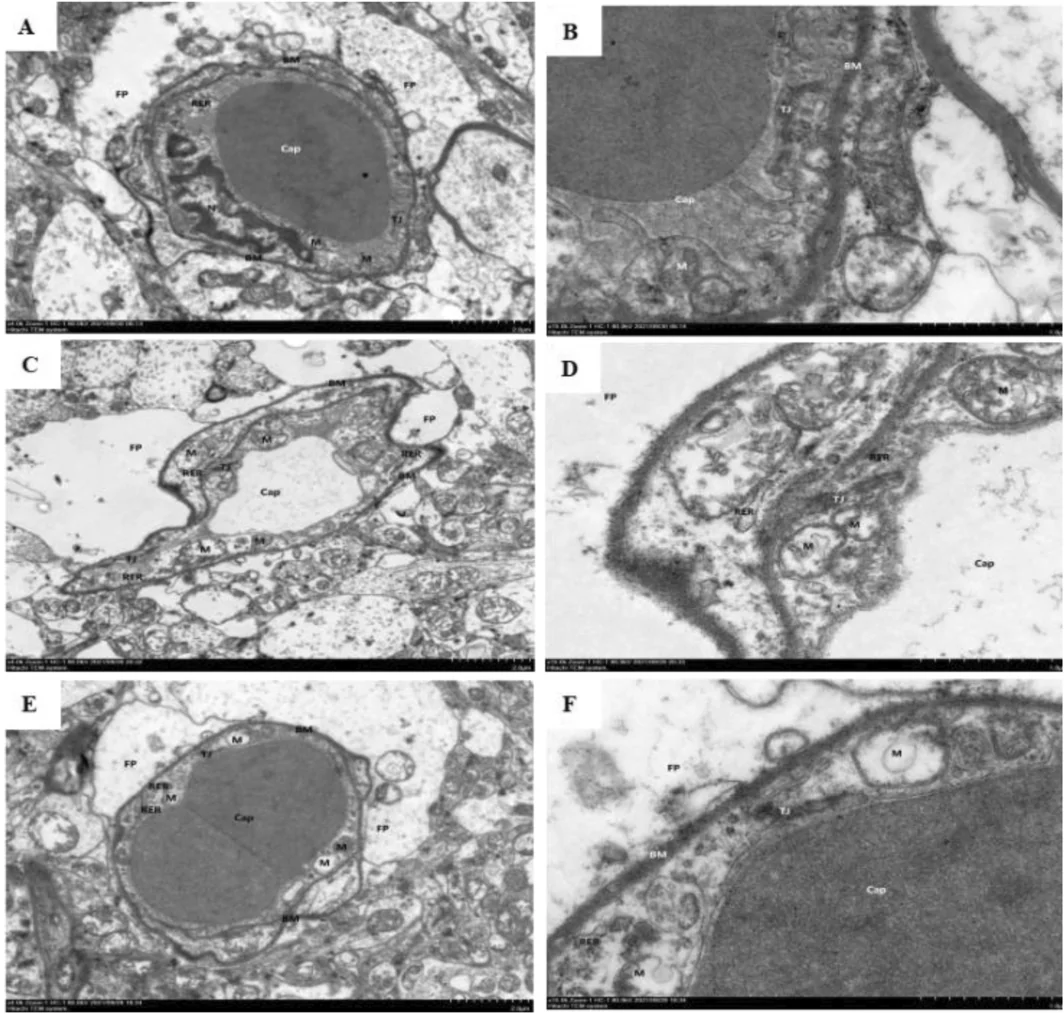
Comparison of TEM in the hippocampus of the PE group over time (A, C, E ×500; B, D, F ×1000). (A) TEM of rat in L‐NAME group at 10 days postpartum (×500). (B) TEM of rat in L‐NAME group at 10 days postpartum (×1000). (C) TEM of rat in L‐NAME group at 1 month postpartum (×500). (D) TEM of rat in L‐NAME group at 1 month postpartum (×1000). (E) TEM of rat in L‐NAME group at 3 months postpartum (×500). (F) TEM of rat in L‐NAME group at 3 months postpartum (×1000). AFP, astrocyte foot process; BBB, blood–brain barrier; Cap, capillary lumen; EC, endothelial cell; Mit, mitochondria; RER, rough endoplasmic reticulum. Black arrows indicate tight junction disruption; red arrows indicate swollen mitochondria; yellow arrows indicate damaged basement membrane.

These findings demonstrate that PE induces chronic and progressive ultrastructural cerebrovascular and neuronal injury in the brain.

## 4 Comment

### 4.1 Principal Findings

Our study employed the Morris water maze test to reveal cognitive dysfunction in a rat model of preeclampsia following parturition, and pravastatin was found to ameliorate these symptoms. Most importantly, MRI can be utilized to assess cognitive dysfunction in the preeclampsia (PE) model rats. Multimodal MRI detected alterations in brain microstructure, microvascular perfusion, and blood–brain barrier in PE model rats, which were consistent with histological and transmission electron microscopic findings. Fractional anisotropy (FA) values in the anterior cortex, posterior cortex, and hippocampus exhibited a negative correlation with escape latencies. The Ktrans values in these brain regions demonstrated a positive correlation with escape latencies. Pravastatin treatment alleviated the pathological changes and cognitive dysfunction in PE model rats to a certain extent, suggesting its potential for the treatment and prevention of PE.

### 4.2 Study Results and Clinical Implications

In this study, high‐dose L‐NAME intraperitoneal injection successfully established a PE model, with hypertension, proteinuria, and increased pup mortality, consistent with previous reports [25]. L‐NAME is a commonly used nitric oxide synthase inhibitor that leads to endothelial dysfunction [26], an important pathogenic mechanism of PE [27]. The imbalance between pro‐angiogenic (PlGF) and anti‐angiogenic factors (sEng) contributes to pathophysiological alterations in advanced disease [28]. On Day 19 of pregnancy, sEng and IL‐6 levels were significantly higher in the PE group than in the NP group, and both were significantly lower after pravastatin treatment. PlGF expression was significantly lower in the PE group and was higher in the PRA group. These findings indicate that L‐NAME induces vascular endothelial dysfunction and systemic inflammation, and pravastatin improves these conditions, consistent with previous studies [29, 30, 31]. PlGF enhances VEGF angiogenic effects [32, 33], and its decrease is seen in placenta‐related diseases [34]. sEng inhibits TGF‐β1‐induced endothelial nitric oxide synthase activation [35]; elevated sEng leads to endothelial dysfunction, vasoconstriction, and adverse maternal/fetal effects [20]. Local or systemic inflammation, with increased TNF‐α and IL‐6, also plays a role in PE [36].

The most effective treatment for PE remains fetal delivery. Low‐dose aspirin after 12 weeks of gestation is recommended for prevention [15], but no drug effectively treats PE and improves prognosis. Our study showed that pravastatin alleviated hypertension, proteinuria, and endothelial dysfunction in PE model rats. Serum sEng decreased significantly after pravastatin treatment, confirming mitigation of endothelial dysfunction. Moreover, pravastatin alleviated cognitive dysfunction, suggesting therapeutic and preventive potential. Pravastatin, a cholesterol‐lowering agent, has gained attention over the past decade [37]. In vitro, pravastatin reduced ET‐1 and SFLT‐1 secretion without toxic effects, supporting its clinical application [38]. Low‐dose pravastatin alleviated oxidative stress caused by placental ischemia in an animal model [39]. Kumasawa et al. [40] reported that pravastatin induced increased PlGF levels in a lentiviral PE model. Some scholars recommend starting pravastatin in the first or second trimester when abnormal angiogenesis is present [41].

Patients with a history of PE may have long‐term cognitive impairments (memory loss, attention deficit, motor speed impairment), associated with PE‐induced brain structural and functional changes. Siepmann et al. reported that a history of PE was associated with white matter abnormalities and cortical volume reduction, with severity proportional to time [42, 43]. Cipolla et al. [44] showed that L‐NAME‐induced hypertension leads to cerebrovascular remodeling and sparse capillaries in the posterior cerebral cortex. Neuroinflammation mediated by glial cells is observed in most neurodegenerative diseases [45]. Olayemi et al. confirmed oligodendrocyte apoptosis in PE mothers during pregnancy and postpartum, proposing this as a cause of long‐term cognitive impairment [4]. Liu et al. [5] reported defects in hippocampal neurogenesis and spatial learning/memory in PE model rats and their offspring. The Morris water maze is widely used to evaluate spatial learning and memory [46, 47]. Previous studies found spatial learning and memory deficits in postpartum PE model rats and their offspring, with abnormal hippocampal neuron and glial cell proliferation [5]. Spatial learning and memory in the water maze task largely depend on the hippocampus and cortical/subcortical regions; prefrontal cortex injury also leads to cognitive impairment [48]. In our study, escape latency was prolonged and platform crossings/time decreased in PE model rats at 10 days, 1 month, and 3 months postpartum, and these metrics worsened over time, indicating progressive postpartum cognitive dysfunction. These indicators improved after pravastatin treatment, demonstrating its therapeutic effect.

DTI is a promising method for characterizing microstructural changes in neuropathology [49]. It provides noninvasive information on white matter microstructure [50]. Decreased FA values reflect microstructural abnormalities [49]. DTI studies have revealed that brain connectivity plays an important role in cognition [51]. In our study, FA values in the anterior cortex, posterior cortex, and hippocampus were significantly lower in the PE group at all postpartum time points, partially recovered after pravastatin treatment, and were significantly negatively correlated with escape latency. HE staining revealed neuronal degeneration and apoptosis in the hippocampus, which worsened over time but were mitigated by pravastatin.

DCE‐MRI is widely used to study BBB disruption in central nervous system diseases, assessing subtle changes in BBB leakage in conditions such as cerebral microvascular disease, diabetes, and Alzheimer's disease [52]. The BBB is a barrier structure composed of endothelial cells, tight junctions, basement membranes, pericytes, and astrocytes; its destruction leads to various CNS diseases [53]. Animal experiments showed that autoregulatory dysfunction of cerebrospinal fluid flow and myogenic tension in PE model rats increases BBB permeability, with morphological evidence of tight junction opening, basement membrane dissolution, and vesicle formation [23]. Wallace et al. [54] reported increased BBB permeability in the posterior cortex, brainstem, and cerebellum of PE model rats, accompanied by postpartum cognitive dysfunction partially mitigated by treatment. Other studies have validated DCE‐MRI measurements of BBB permeability against gold‐standard histology in stroke models [55]. Extracellular vesicles from PE patient plasma damaged the BBB in mice, suggesting a mechanism for cerebral complications of PE [56]. Another study found higher IL‐6 and IL‐8 levels in the cerebrospinal fluid of PE patients, indicating neuroinflammation and BBB damage [57]. In our study, Ktrans values increased in PE model rats postpartum, TEM images confirmed BBB damage, and cognitive dysfunction was observed. Correlation analysis showed a positive correlation between Ktrans and escape latency.

IVIM is an advanced diffusion‐weighted imaging method that uses multiple *b*‐values to distinguish water diffusion from tissue perfusion without exogenous contrast. The pseudodiffusion coefficient (D* value) reflects microcapillary perfusion [58]. IVIM‐related indicators in ischemic stroke are highly consistent with perfusion‐weighted imaging [59]. A previous study found decreased cerebral blood volume and flow in the caudate nucleus of third‐trimester PE patients using IVIM [60]. In AD patients, D* values decreased in some brain regions and were positively correlated with cognitive impairment severity, suggesting IVIM parameters as biomarkers [61]. Our study revealed that D* values in the PE group were generally lower than controls and decreased further over time, indicating reduced cerebral microcirculation perfusion; these changes were partially abrogated by pravastatin.

### 4.3 Strengths and Limitations

The strengths of this study are as follows. First, we used intraperitoneal injection of 250 mg/kg L‐NAME, whereas previous literature predominantly used L‐NAME in drinking water or subcutaneous injection [62]. The commonly used intraperitoneal dose ranges from 50 to 150 mg/(kg·day); we increased the dose to 250 mg/(kg·day) to enhance modeling success. Pups in the model group exhibited a higher incidence of limb disabilities (predominantly hindlimb deficiencies), and the average number and body weight of surviving pups were significantly reduced. Higher L‐NAME dosage exaggerates placental ischemia, angiogenic imbalance, and systemic inflammatory responses, which are major contributors to adverse fetal outcomes. This design strengthens the model's clinical relevance to severe PE and facilitates the study of long‐term neurological outcomes. Secondly, in addition to cross‐sectional comparisons, we conducted longitudinal comparisons at multiple time points within each group (tail artery blood pressure, serum sEng, PlGF, SFlt‐1, IL‐6, and E2 on GD19, 10 days, 1 month, and 3 months postpartum; water maze tests; multimodal MRI). Model group rats underwent brain transmission electron microscopy, and all groups underwent HE and Nissl staining. These data allowed both cross‐sectional assessment of modeling success and treatment efficacy, as well as longitudinal observation of changes in blood pressure, endothelial function, and cognitive function. Third, we used multiple multimodal MRI indicators to evaluate brain structure and function in PE rats and performed correlation analyses with cognitive function, finding correlations between FA, Ktrans, and cognitive function. Lastly, pravastatin exhibits antioxidant stress and vascular endothelial protective effects, which mechanistically benefit PE prevention and treatment. Our study found that pravastatin significantly reduced L‐NAME‐induced hypertension, urinary albumin/creatinine, protected endothelial function, decreased serum sEng, SFlt‐1, and IL‐6, increased PlGF levels, and improved cognitive dysfunction. However, a limitation is the inadequate sample size (only 7 rats per subgroup), which may necessitate validation with a larger sample size.

Another limitation of this study is the absence of a normal pregnant group treated with pravastatin (NP + Pravastatin), meaning that the specificity of pravastatin's therapeutic effects on PE‐associated cognitive dysfunction cannot be fully distinguished from potential non‐specific effects. Additionally, the absence of quantitative morphometric analysis for HE and Nissl staining further limits the study; although representative histopathological images clearly demonstrate neuronal degeneration and Nissl body loss in the PE group, the lack of quantitative data (e.g., neuronal density counts or integrated optical density of Nissl bodies) reduces the robustness of our histological conclusions. Future studies incorporating both an NP + Pravastatin group and systematic stereological or ImageJ‐based quantification are warranted to address these issues and validate our findings.

## 5 Conclusion

In summary, our data indicate that MRI can be used to evaluate the cognitive dysfunction in PE model rats. Changes in brain microstructure, microvascular perfusion and the BBB detected via multimodal MRI in PE model rats corresponded to the histology and transmission electron microscopy results. FA values in the anterior cortex, posterior cortex and hippocampus were negatively correlated with escape latency. The Ktrans values in these areas were positively correlated with escape latency. Pravastatin treatment can alleviate the pathological changes and cognitive dysfunction of PE model rats to a certain extent, suggesting its potential as a therapeutic agent for PE. However, the absence of a normal pregnancy + pravastatin control group in the current study precludes definitive conclusions regarding the specificity of this effect, and further studies may provide new hope for the prevention and treatment of PE.

## Author Contributions

Bo Gao conceived the study design and oversaw all animal experiments. Yong Xia collected experimental data and edited the manuscript. Junguo Zhao conducted statistical analyses, interpreting results to draw scientific conclusions.

## Funding

This work is supported by the National Natural Science Foundation of China (81871333, 82260340), Guizhou Province Science & Technology Project ([2020]4Y159, [2021]430), Discipline Leading Talent of The Affiliated Hospital of Guizhou Medical University (gyfyxkrc‐2023‐04), and 942 plan for Guizhou Province Key Clinical Specialties (GZWJWPF2025017).

## Conflicts of Interest

The authors declare no conflicts of interest.

## Data Availability

The data that support the findings of this study are available from the corresponding author upon reasonable request.
